# Digital Problem-Based Learning in Health Professions: Systematic Review and Meta-Analysis by the Digital Health Education Collaboration

**DOI:** 10.2196/12945

**Published:** 2019-02-28

**Authors:** Lorainne Tudor Car, Bhone Myint Kyaw, Gerard Dunleavy, Neil A Smart, Monika Semwal, Jerome I Rotgans, Naomi Low-Beer, James Campbell

**Affiliations:** 1 Family Medicine and Primary Care Lee Kong Chian School of Medicine Nanyang Technological University Singapore Singapore; 2 Department of Primary Care and Public Health School of Public Health Imperial College London London United Kingdom; 3 Centre for Population Health Sciences Lee Kong Chian School of Medicine Nanyang Technological University Singapore Singapore; 4 School of Science & Technology University of New England Armidale Australia; 5 Medical Education Research Unit Lee Kong Chian School of Medicine Nanyang Technological University Singapore Singapore; 6 Health Workforce Department World Health Organization Geneva Switzerland

**Keywords:** randomized controlled trials, effectiveness, systematic review, problem-based learning, medical education

## Abstract

**Background:**

The use of digital education in problem-based learning, or digital problem-based learning (DPBL), is increasingly employed in health professions education. DPBL includes purely digitally delivered as well as blended problem-based learning, wherein digital and face-to-face learning are combined.

**Objective:**

The aim of this review is to evaluate the effectiveness of DPBL in improving health professionals’ knowledge, skills, attitudes, and satisfaction.

**Methods:**

We used the gold-standard Cochrane methods to conduct a systematic review of randomized controlled trials (RCTs). We included studies that compared the effectiveness of DPBL with traditional learning methods or other forms of digital education in improving health professionals’ knowledge, skills, attitudes, and satisfaction. Two authors independently screened studies, extracted data, and assessed the risk of bias. We contacted study authors for additional information, if necessary. We used the random-effects model in the meta-analyses.

**Results:**

Nine RCTs involving 890 preregistration health professionals were included. Digital technology was mostly employed for presentation of problems. In three studies, PBL was delivered fully online. Digital technology modalities spanned online learning, offline learning, virtual reality, and virtual patients. The control groups consisted of traditional PBL and traditional learning. The pooled analysis of seven studies comparing the effect of DPBL and traditional PBL reported little or no difference in postintervention knowledge outcomes (standardized mean difference [SMD] 0.19, 95% CI 0.00-0.38). The pooled analysis of three studies comparing the effect of DPBL to traditional learning on postintervention knowledge outcomes favored DPBL (SMD 0.67, 95% CI 0.14-1.19). For skill development, the pooled analysis of two studies comparing DPBL to traditional PBL favored DPBL (SMD 0.30, 95% CI 0.07-0.54). Findings on attitudes and satisfaction outcomes were mixed. The included studies mostly had an unclear risk of bias.

**Conclusions:**

Our findings suggest that DPBL is as effective as traditional PBL and more effective than traditional learning in improving knowledge. DPBL may be more effective than traditional learning or traditional PBL in improving skills. Further studies should evaluate the use of digital technology for the delivery of other PBL components as well as PBL overall.

## Introduction

Problem-based learning (PBL) has been used as an educational approach in health professions education in many medical and nursing school curricula worldwide for over 50 years [1]. PBL aims to foster a wide range of skills such as communication and collaboration skills, decision making, problem solving, critical thinking, and self-directed learning [2]. In PBL, the use of real, ill-structured problems provides a context for the development of students’ knowledge and skills [3]. Learning in PBL is student centered and occurs in small collaborative groups while teachers take on the role of tutors. Although its implementation varies across different settings, PBL, in general, is an iterative process consisting of three parts: a problem-presentation and analysis phase, a self-directed learning phase, and a synthesis and reporting phase [4].

Worldwide, various components of PBL are being increasingly delivered using digital technology. Digital education is changing the way in which health professions education, including PBL, is conducted. Digital education may comprise a variety of interventions based on learning tools, theories, content, objectives, teaching methods, and setting of delivery. In terms of the type of learning technologies, digital education includes, but is not restricted to, online and offline computer-based learning, massive open online courses, virtual reality, virtual patient simulation, mobile learning, serious gaming and gamification, and psychomotor skills trainers (Multimedia Appendix 1) [5-16]. Studies on the use of digital technologies in health professions education, in general, have reported its advantages over traditional learning in terms of improved diagnostic reasoning skills, interpersonal and professional competencies, long-term knowledge retention, problem-solving skills, self-direct/lifelong learning skills, higher-order thinking skills, self-perception, and confidence [3,17-23]. Although there is evidence on different applications of digital technology in PBL, it is still unclear how effective it is to integrate digital technology within PBL as compared to traditional PBL [23].

Digital problem-based learning (DPBL), or the use of different types of digital technologies to deliver PBL, has the potential to enhance the authenticity, appeal, accessibility, and effectiveness of PBL by enhancing participants’ communication, collaboration and self-learning [24-26]. DPBL includes both fully digitally delivered PBL as well as blended PBL, wherein digital education is used to deliver certain components of PBL, while the rest of it is delivered face to face. Although there are reviews on diverse ways that technology can be incorporated in PBL, the evidence on the effectiveness of its use in PBL in health professions education is lacking [23,27]. Our objective in this review was to evaluate the effectiveness, economic impact, and potential adverse effects of DPBL interventions compared to other forms of learning in health professions education.

## Methods

### Study Selection

We followed the Cochrane methodology for every step of the review [28]. A detailed description of the methodology has been previously provided by the Digital Health Education collaboration [29]. The Digital Health Education collaboration is an international initiative evaluating the effectiveness of digital education in health professions education through a series of methodologically robust systematic reviews.

In this review, we included randomized controlled trials (RCTs) that evaluated the effectiveness of DPBL in improving health professionals’ knowledge, skills, attitudes, and satisfaction of students and compared DPBL with traditional learning methods or other forms of digital learning. Crossover trials were excluded because of a high likelihood of a carry-over effect.

We included studies with preregistration as well as postregistration health professionals as per the qualifications listed in the Health Field of Education and Training (091) of the International Standard Classification of Education [30]. However, we excluded students of alternative, traditional, and complementary medicine. We excluded studies that focused on hybrid PBL (ie, a combination of PBL and traditional learning approaches).

We included studies in which any form of digital technology was used in combination with PBL for delivering the learning content of courses, either as the sole (full digital learning) or partial (blended learning) means of delivery, for the purpose of learning in health professions education. Digital technology primarily supports PBL principles and processes by enabling contextual and collaborative learning [27]. We defined traditional PBL in line with the Maastricht model as small-group, self-directed, tutor-supported learning that revolves primarily around a problem and occurs face to face [31]. We further conceptualized the role of digital technology in PBL in line with the presented framework that builds on the Maastricht PBL framework and the Arena Blended Connected (ABC) curriculum design method [32-34]. The Maastricht PBL framework differentiates among three broad components in PBL: the first meeting, self-directed learning, and the second meeting [34]. These components are present in traditional PBL delivered face to face. Each of these three components encompasses different learning activities that we outline using the ABC curriculum design approach. The ABC curriculum design method differentiates six main learning activities that can be supported with the use of digital technology: acquisition, inquiry, practice, production, discussion, and collaboration [33]. In our framework, we present examples of how different digital tools can be employed in a variety of ways to support learning activities in PBL. For example, learning activities comprised in the first PBL component include acquisition, discussion, collaboration, practice, and investigation. Digital technology can be used in various ways to deliver each of these learning activities. For instance, acquisition of information can be achieved through the use of multimedia resources, podcasts, or text messages. Collaboration can be supported through the use of chatrooms or Web forums. Practice, on the other hand, can be facilitated via digital education modalities that support simulation such as virtual reality or virtual patients.

We analyzed studies that compared DPBL to traditional PBL or traditional learning (textbook, lectures, etc) and to different forms of DPBL interventions or other digital education.

We excluded studies that focused on individual learning interventions, evaluated the use of DPBL in other educational areas, lacked an active comparison, and assessed interventions with optional or minimal use of digital technology.

We included the following primary outcomes: (1) students’ postintervention cognitive knowledge and skills measured with any instrument (validated or nonvalidated); (2) students’ professional postintervention attitudes toward DPBL interventions, patients, or new clinical knowledge or skills measured using any instruments (validated or nonvalidated); students’ postintervention satisfaction with DPBL intervention, measured using any instrument (validated or nonvalidated).

For secondary outcomes, we focused on the economic impact of the DPBL intervention and potential adverse or unintended effect of the DPBL intervention.

### Data Sources, Collection, and Quality Assessment

We searched seven electronic databases, namely, MEDLINE (Ovid), Embase (Elsevier), Cochrane Central Register of Controlled Trials (CENTRAL, Wiley), PsycINFO (Ovid), Educational Research Information Centre (Ovid), Cumulative Index to Nursing and Allied Health Literature (Ebsco), and Web of Science Core Collection (Thomson Reuters), for relevant studies from January 1990 to August 16, 2017, without language restrictions (Multimedia Appendix 2).

We also checked the reference lists of all included studies and relevant systematic reviews and searched the International Clinical Trials Registry and metaRegister of Controlled Trials for unpublished trials. We followed the Cochrane methodology for the selection of studies, data extraction, data analysis, and risk of bias analysis, with two reviewers independently performing each of these steps [28]. From each study, the following information was extracted: first author’s surname, publication year, course name, sample size, student characteristics, intervention method, duration of study, and outcomes. We contacted the study authors for missing data and assessed the risk of bias in the included studies using the Cochrane risk-of-bias tool [28].

### Data Analysis

Standardized mean difference (SMD) for continuous outcomes, with 95% CI, was calculated based on the availability of data from the included studies. We pooled studies together based on comparison and outcomes using SMD. We interpreted the effect size using the Cohen rule of thumb (ie, with <0.2 representing no effect, 0.2 to <0.5 representing a small effect, 0.5 to <0.8 representing a moderate effect, and ≥0.8 representing a large effect) [28,35]. We employed a random-effects model in our meta-analysis. The I^2^ statistic was employed to assess heterogeneity, with I^2^<25%, 25%-75%, and >75% representing a low, moderate, and high degree of inconsistency, respectively. The meta-analysis was performed using Review Manager 5.3 (Cochrane Library Software, Oxford, UK). We reported the findings in line with the Preferred Reporting Items for Systematic Reviews and Meta-Analyses reporting standards. We present the findings that we were unable to pool due to lack of data or high heterogeneity, in the form of a narrative synthesis.

## Results

### Search Results

**Figure 1:**
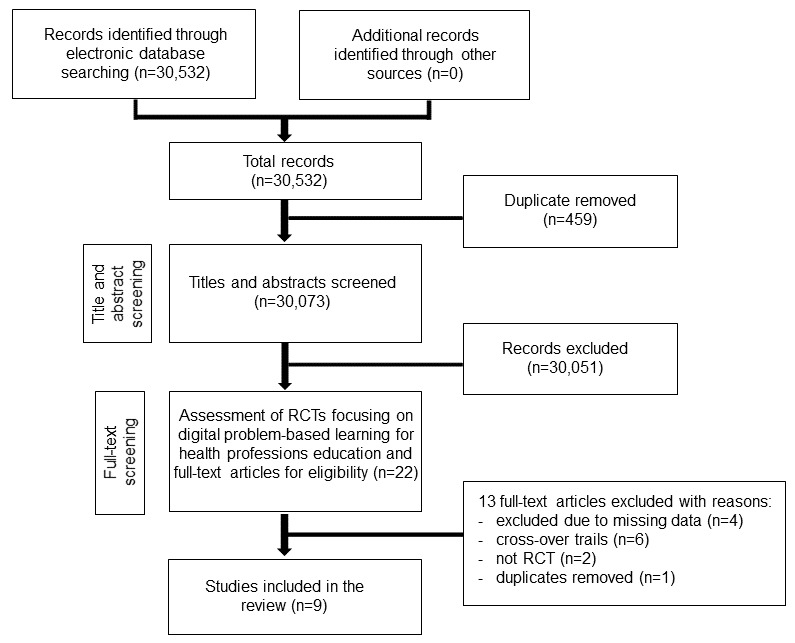
Preferred Reporting Items for Systematic Reviews and Meta-Analyses flow diagram. RCT: randomized controlled trial.

The search strategy yielded 30,532 references. We included nine studies with 890 medical students (Figure 1). We excluded four studies due to missing data [20,36-38]. No relevant ongoing clinical trials were identified.

### Study Characteristics

The characteristics of the nine included studies are presented in Table 1. All studies were RCTs and published in English. Seven studies were conducted in high-income countries and two studies, in middle-income countries [21,31]. Five studies had two arms, one study had three arms, and three studies had four arms. Four studies exclusively compared DPBL to traditional PBL, one study exclusively compared DPBL to traditional learning, and two studies compared DPBL to both traditional PBL and traditional learning [21,31]. None of the included studies compared different forms of DPBL. The fields of study varied across the included studies. The studies focused on pregnancy-associated urinary incontinence [39], biochemistry (acid-based physiology) [40], genetics [41], internal medicine [42], ophthalmology [21], dermatology [31], multidisciplinary [43], human physiology [22], and traumatic head injury [44].

**Figure 2:**
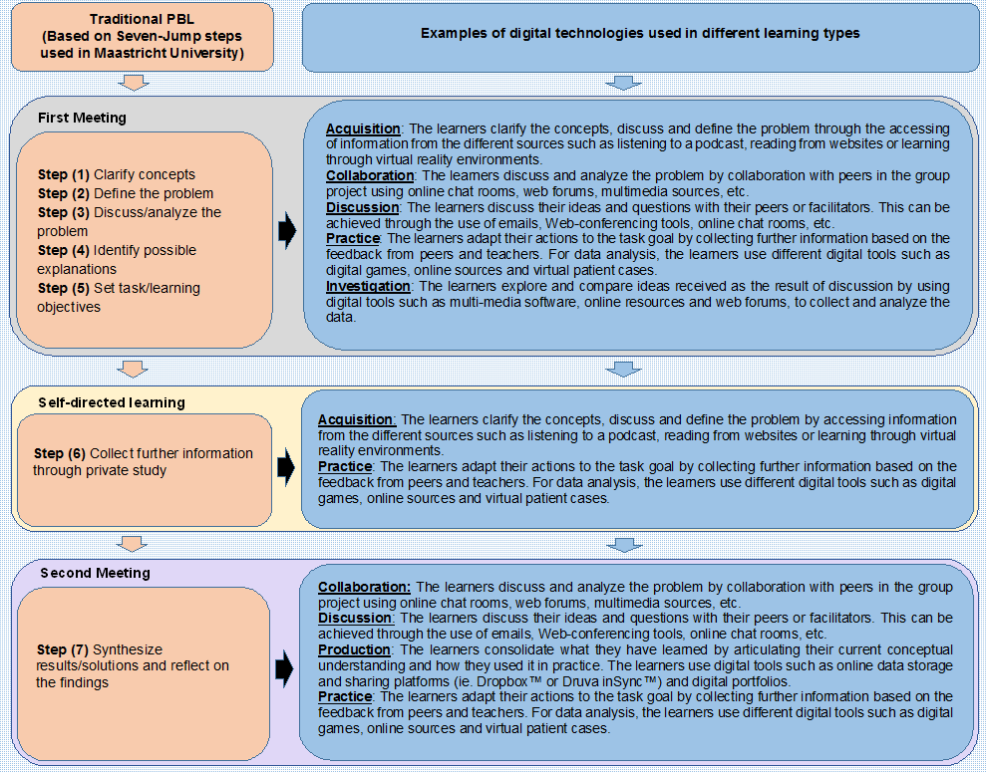
A conceptual framework for the use of digital technology in PBL. PBL: problem-based learning.

In most studies, DPBL interventions were delivered face to face and digital technology was employed for one component of the PBL process—presentation of problems. In four studies, DPBL was at least partially delivered on a distance basis. In one study, DPBL was employed for delivery of the initial part of the PBL (ie, the first meeting; Figure 2) [43]. In three studies, DPBL was fully distance based and all components of PBL were delivered using digital technology [39,40,44]. In line with our framework, in studies with digital presentation of problems, digital technology was mostly used to support acquisition (Figure 2; Multimedia Appendix 3). In fully distance-based DPBL, digital technology enabled a range of learning activities such as acquisition, investigation, collaboration, discussion, and production. In three studies, digital technology allowed students to practice through the use of virtual reality and virtual patients [22,41,42].

The types of digital education modalities included offline learning (eg, CD-ROM) [31], online learning (eg, multimedia modules) [21,22,39,40,43,44], (immersive) virtual reality [41], and virtual patients [42] (Table 1). The control groups used traditional PBL such as text-based or paper-based PBL in six studies [22,39,40,42-44], traditional PBL or traditional learning in two studies [21,31], and solely traditional learning in one study [41]. The duration of the interventions ranged from 1 hour 33 minutes [41] to 7 weeks [31]. One study did not report the duration of the intervention [43].

**Figure 3:**
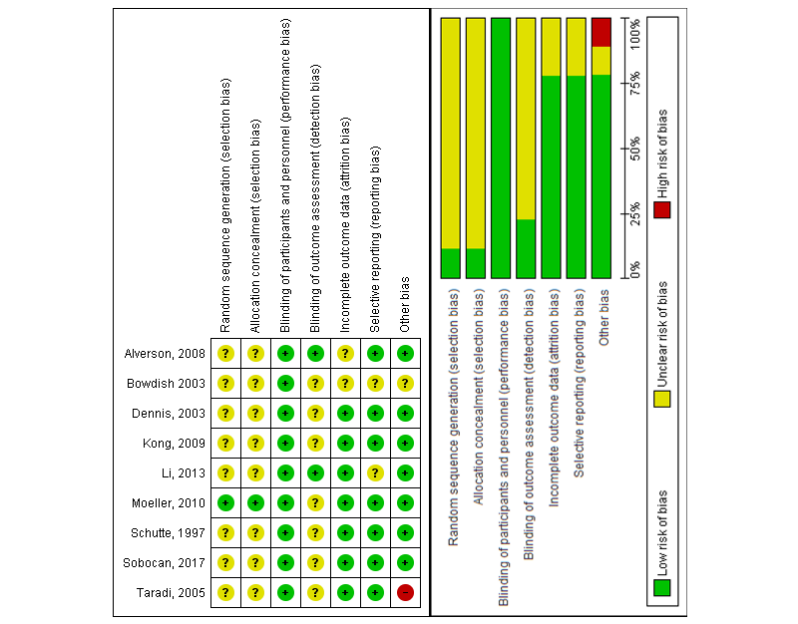
Risk-of-bias summary: review authors' judgement about each risk-of-bias item for each included study.

Included studies reported findings on students’ knowledge, skills, attitude, and satisfaction. No studies reported cost-related outcomes or adverse/unintended effects of DPBL-based interventions. All studies measured outcomes immediately after the intervention, except Sobocan et al [42], who reported both immediate and long-term knowledge retention at the end of the academic year. The included studies mostly had an unclear risk of bias due to a lack of information on randomization, allocation concealment, and blinding of outcomes assessment (Figure 3).

### Effects of Digital Problem-Based Learning Versus Traditional Problem-Based Learning

**Figure 4:**
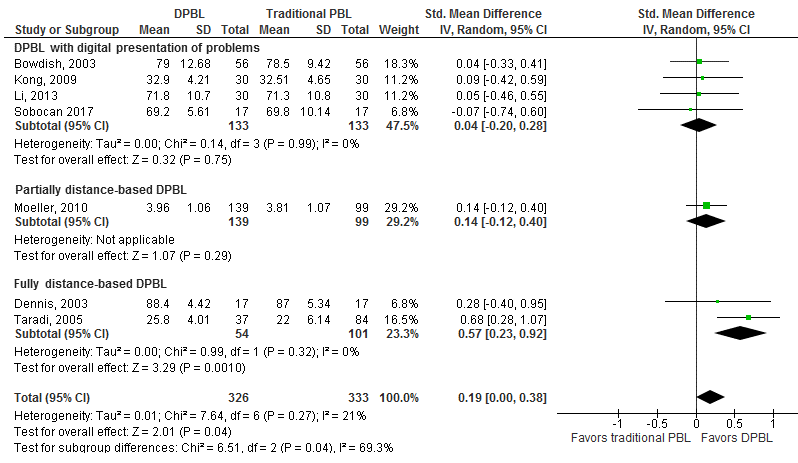
The effect of DPBL compared to traditional PBL (knowledge outcome, postintervention). DPBL: digital problem-based learning; PBL: problem-based learning; IV: interval variables, Random: random effect model.

The effects of DPBL compared to the traditional PBL on knowledge scores were reported in eight studies involving 822 medical students (Multimedia Appendix 4) [21,22,31,39,40,42-44]. The pooled analysis of seven studies showed little or no difference between DPBL and traditional PBL in postintervention knowledge scores (SMD 0.19, 95% CI 0.00-0.38; DPBL group, n=326; traditional PBL group, n=333; moderate quality; Figure 4) [21,22,31,39,40,42,43]. One study assessed the long-term effects on knowledge and reported no difference between the groups.

We also performed subgroup analysis based on the degree to which the digital technology was employed as part of PBL. We differentiated among studies in which digital technology was used for presentation of problems [21,22,31,42], the first part of the PBL was distance based [43], and PBL was fully distance based [39,40]. We found a statistically significant difference among these subgroups. There was a moderate improvement in postintervention knowledge scores in fully distance-based DPBL compared to traditional PBL (SMD 0.57, 95% CI 0.23-0.92) and no difference in studies on DPBL with digital presentation of problems (Figure 4).

**Table 1:** Characteristics of the included studies.

Comparisons groups and studies		Learning modalities compared	Number and types of participants	Field of study	Outcomes
**DPBL^a^ vs traditional PBL^b^**					
	Alverson et al 2008 [44], RCT^c^, United States	VR^d^ PBL vs traditional PBL	36 medical students (year unspecified)	Traumatic head injury	Knowledge
	Bowdish et al 2003 [22], RCT, United States	Online PBL vs traditional PBL	150 medical students (first year)	Human physiology	Knowledge
	Dennis 2003 [39], RCT, United States	Online PBL vs traditional PBL	34 medical students (second year)	Pregnancy-associated urinary incontinence	Knowledge
	Kong et al 2009 [21], RCT, China	Online PBL vs traditional PBL	90 medical students (year unspecified)	Ophthalmology	Knowledge
	Li et al 2013 [31], RCT, China	Offline PBL vs traditional PBL	120 medical students (fourth year)	Dermatology	Knowledge, Skills
	Moeller et al 2010 [43], RCT, Germany	Online PBL vs traditional PBL	237 medical students (year unspecified)	Multidisciplinary	Knowledge, Skills
	Sobocan et al 2017 [42], RCT, Slovenia	VP^e^-based PBL vs traditional PBL	34 medical students (third year)	Internal medicine	Knowledge
	Taradi et al 2005 [40], RCT, Croatia	Online PBL vs traditional PBL	121 medical students (second year)	Biochemistry (acid-base physiology)	Knowledge
**DPBL vs traditional learning**					
	Kong et al 2009 [21], RCT, China	Online PBL vs traditional learning (lecture)	90 medical students (year unspecified)	Ophthalmology	Knowledge
	Li et al 2013 [31], RCT, China	Offline PBL vs traditional learning (lecture)	120 medical students (fourth year)	Dermatology	Knowledge
	Schutte et al 1997 [41], RCT, The Netherlands	VR PBL vs traditional learning (textbook)	68 medical students (first year)	Genetics (global structure of DNA)	Knowledge

^a^DPBL: digital problem-based learning.

^b^PBL: problem-based learning.

^c^RCT: randomized controlled trial.

^d^VR: virtual reality.

^e^VP: virtual patient.

The effects of DPBL compared to the traditional PBL on skills scores were reported in two studies (N=357). The pooled analysis of these two studies showed that DPBL may slightly improve postintervention skill scores (SMD 0.30, 95% CI 0.07-0.54; *P*=.01) in comparison to traditional PBL [31,43].

The effects of DPBL compared to the traditional PBL on satisfaction scores were reported in three studies with mixed findings. Two studies evaluating the use of DPBL with digitally presented problems reported no difference between DPBL and traditional PBL in satisfaction scores [21,31]. One study reported a significant difference in satisfaction scores in favor of fully digitally delivered, distance-based PBL [40]. Two studies (N=126) assessed students’ attitude toward the intervention and reported mixed results or incomplete outcome data [21,44].

### Effects of Digital Problem-Based Learning Versus Traditional Learning

**Figure 5:**
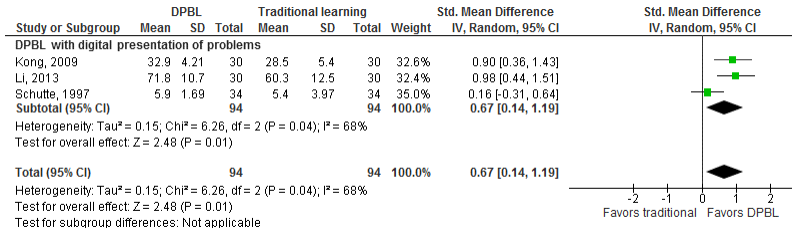
The effect of DPBL compared to traditional learning (knowledge outcome, postintervention). IV: interval variables; Random: random effect model; DPBL: digital problem-based learning.

The effects of DPBL compared to traditional learning on knowledge scores were reported in three studies (N=278) [21,31,41] (Multimedia Appendix 4). The pooled analysis of three studies showed that DPBL may moderately improve postintervention knowledge scores (SMD 0.67, 95% CI 0.14-1.19) in DPBL compared to traditional teaching (Figure 5). A subgroup analysis of two studies (N=210) evaluating DPBL with digital presentation of problems and traditional learning showed a large improvement in postintervention knowledge scores (SMD 0.94, 95% CI 0.56-1.31) [21,31].

The effects of DPBL compared to traditional learning on skills scores were reported in one study. This study [31] reported higher postintervention skill scores in the DPBL group than in the traditional learning group (SMD 1.13, 95% CI 0.58-1.67). The effects of DPBL compared to traditional learning on satisfaction scores were reported in two studies (N=210) with uncertain findings (SMD 0.73, 95% CI –0.17 to 1.63).

## Discussion

### Overview

In this review, we evaluated the effectiveness of the use of digital technology for delivering PBL. Our findings show that DPBL improves students’ postintervention knowledge scores in comparison to traditional learning. DPBL is as effective as traditional PBL in improving students’ postintervention knowledge and may slightly improve postintervention skills. Moreover, fully digitally delivered, distance-based DPBL may lead to better knowledge scores in comparison to traditional PBL. The risk of bias in the included studies was mostly judged as unclear due to a lack of information on randomization, allocation concealment, and outcome assessment blinding. In the included studies, the term “blended PBL” was employed to denote diverse configurations of digital technology and PBL. For example, an intervention in which PBL was fully delivered online, but included one visit to the clinic, and another intervention in which face-to-face delivered PBL included digital presentation of problems were both termed blended learning. The use of “blended learning” therefore seemed misleading in this context. We decided to focus primarily on describing the way in which digital technology was employed in the PBL process. From our viewpoint, there are two main applications of digital technology in PBL: full or partial delivery of distance-based PBL or support of delivery of different components of face-to-face or colocated PBL. Most studies included in our review focused on colocated PBL, with digital technology used for the presentation of problems. This corresponds to the findings from two reviews focusing on the application of digital technology in PBL [23,27]. The reviews also report that in most studies, digital technology was used to provide contextual learning and present problems. One of these reviews focused on PBL from all disciplines and also highlighted the common use of digital technology for collaborative learning as part of distance-based PBL. In our review, we have found limited evidence on distance-based PBL in health professions education. There is a need for more research on this type of DPBL and to explore other applications of digital technology in face-to-face PBL, such as supporting collaboration, discussion, investigation, and practice. We present suggestions for the diverse applications of digital tools in PBL in our framework in Figure 2.

Our findings show that DPBL was more effective than traditional learning. Although we were unable to find meta-analyses comparing DPBL to other forms of education, there are numerous meta-analysis comparing traditional PBL to traditional learning [45-49]. The more recently published ones report that PBL is more effective than traditional learning, with moderate-to-large improvement in knowledge [45,46]. In our study, we found only three studies comparing DPBL and traditional learning, which reported an overall moderate improvement in postintervention knowledge scores among DPBL learners. A small subgroup analysis of studies in which digital technology was employed for the presentation of problems showed a major improvement in knowledge in the DPBL groups compared to the traditional learning groups. This may indicate that different configurations of DPBL may lead to larger knowledge gains. There is a need for more studies comparing the effect of distance-based DPBL or the use of digital technology for the support of other PBL components to the effect of traditional learning.

We found that DPBL was as effective as PBL in terms of knowledge, and fully digitally delivered distance-based DPBL was potentially more effective than traditional PBL. Although there are some nonrandomized studies on distance-based PBL corroborating this finding, the evidence from RCTs is scarce [23]. Potential reasons for the greater effectiveness of distance-based PBL include greater student interaction, involvement, and engagement among students. We also found that DPBL may be more effective than PBL for skills while satisfaction outcome data were mixed. These findings are based on a small number of studies, primarily assessing short-term effectiveness. More research is needed to evaluate the effectiveness of different configurations of digitally supported and distance-based PBL.

Our review has several limitations. Although RCTs provide the highest level of evidence for the effectiveness of an intervention, it is not always possible to use a rigorous RCT approach in educational research [50]. Studies included in this meta-analysis were designed as RCT, but most of them lacked information on the randomization method, allocation concealment, or blinding method. Furthermore, they mostly reported solely postintervention data; therefore, we could not calculate the pre-post intervention change. We assumed groups were matched at baseline for key characteristics and outcome measure scores. There was no information on DPBL in postregistration health professionals. Moreover, there was limited or no information on other outcomes such as skills, attitudes, satisfaction, costs, and adverse/untoward effects of DPBL. The included studies assessed short-term effectiveness, with only one study reporting a follow-up assessment. Strengths of our review include a comprehensive and sensitive search; clear inclusion and exclusion criteria encompassing a broad range of students, outcomes, and interventions; extraction of duplicate, independent, and reproducible data; and rigorous assessment of the risk of bias.

### Conclusions

DPBL includes diverse applications of digital technology as part of face-to-face as well as distance-based PBL. Our findings suggest that DPBL is more effective than traditional learning and as effective as traditional PBL in improving postintervention knowledge outcomes. For improvement of skill outcomes, DPBL may be more effective than traditional learning or traditional PBL. There is limited evidence for other outcomes such as satisfaction, attitudes, cost effectiveness, and adverse effects. Most studies evaluated the use of digital technology for the presentation of problems as part of face-to-face DPBL and had unclear risk of bias. There is scope for the evaluation of digital technology in the delivery of other PBL components as well as the effectiveness of distance-based PBL.

## Appendix

Multimedia Appendix 1 Description of the digital education modalities.

Multimedia Appendix 2 MEDLINE (Ovid) search strategy.

Multimedia Appendix 3 Digital technologies used in the studies and associated learning types.

Multimedia Appendix 4 Characteristics and effects of the included studies.

## Abbreviations

ABC: Arena Blended Connected
DPBL: digital problem-based learning
SMD: standardized mean difference
PBL: problem-based learning
RCT: randomized controlled trial
VP: virtual patient
VR: virtual reality

## References

[1] Lim WK (2012). Dysfunctional problem-based learning curricula: resolving the problem. BMC Med Educ.

[2] Wilder S (2014). Impact of problem-based learning on academic achievement in high school: a systematic review. Educational Review.

[3] Reich S, Simon JF, Ruedinger D, Shortall A, Wichmann M, Frankenberger R (2007). Evaluation of two different teaching concepts in dentistry using computer technology. Adv Health Sci Educ Theory Pract.

[4] Yew EHJ, Goh K (2016). Problem-Based Learning: An Overview of its Process and Impact on Learning. Health Professions Education.

[5] Hervatis V, Kyaw BM, Semwal M, Dunleavy G, Tudor Car L, Zary N (2016). PROSPERO CRD42016045679.

[6] Saxena N, Kyaw BM, Vseteckova J, Dev P, Paul P, Lim KTK, Kononowicz AA, Masiello I, Tudor Car L, Nikolaou CK, Zary N, Car J (2016). PROSPERO CRD42016045470.

[7] Tudor Car L, Riboli-Sasco E, Belisario JM, Nikolaou CK, Majeed A, Zary N, Car J (2015). PROSPERO CRD42015029786.

[8] Gentry S, Gauthier A, L'Estrade Ehrstrom B, Wortley D, Lilienthal A, Tudor Car L, Dauwels-Okutsu S, Nikolaou CK, Zary N, Campbell J, Car J (2019). Serious gaming and gamification education in health professions: a systematic review by the Digital Health Education Collaboration. J Med Internet Res (forthcoming).

[9] Dunleavy G, Nikolaou CK, Nifakos S, Atun R, Law GCY, Tudor Car L (2019). Mobile Digital Education for Health Professions: Systematic Review and Meta-Analysis by the Digital Health Education Collaboration. J Med Internet Res.

[10] Huang Z, Semwal M, Lee SY, Tee WKM, Ong W, Tan WS, Bajpai R, Tudor Car L (2019). Digital health professionals? diabetes management education and training: a systematic review by the Digital Health Education collaboration. J Med Internet Res (forthcoming).

[11] Kyaw BM, Saxena N, Posadzki P, Vseteckova J, Nikolaou CK, George PP, Divakar U, Masiello I, Kononowicz AA, Zary N, Tudor Car L (2019). Virtual Reality for Health Professions Education: Systematic Review and Meta-Analysis by the Digital Health Education Collaboration. J Med Internet Res.

[12] Posadzki P, Bala M, Kyaw BM, Semwal M, Divakar U, Koperny M, Sliwka A, Car J (2019). Offline digital education for post-registration health professions: a systematic review by the Digital Health Education collaboration. J Med Internet Res (forthcoming).

[13] Semwal M, Whiting P, Bajpai R, Bajpai S, Kyaw BM, Tudor Car L (2019). Digital education for health professions on smoking cessation management: a systematic review by the Digital Health Education collaboration. J Med Internet Res (forthcoming).

[14] Posadzki P, Paddock S, Campbell J, Car J (2018). Medical students' digital education of communication skills: a systematic review by the Digital Health Education Collaboration. J Med Internet Res (forthcoming).

[15] George PP, Zhabenko O, Kyaw BM, Antoniou P, Posadzki P, Saxena N, Semwal M, Tudor Car L, Zary N, Lockwood C, Car J (2019). Medical doctors' online digital education: a systematic review by the Digital Health Education Collaboration. J Med Internet Res (forthcoming).

[16] Wahabi H, Esmaeil SA, Bahkali KH, Titi MA, Amer YS, Fayed AA, Jamal A, Zakaria N, Siddiqui AR, Semwal M, Tudor Car L, Posadzki P, Car J (2019). Medical doctors' offline-computer-assisted digital education: a systematic review by the Digital Health Education Collaboration. J Med Internet Res (forthcoming).

[17] Al-Dahir S, Bryant K, Kennedy KB, Robinson DS (2014). Online virtual-patient cases versus traditional problem-based learning in advanced pharmacy practice experiences. Am J Pharm Educ.

[18] Chan DH, Leclair K, Kaczorowski J (1999). Problem-based small-group learning via the Internet among community family physicians: a randomized controlled trial. MD Comput.

[19] Kim JH, Shin JS (2014). Effects of an online problem-based learning program on sexual health care competencies among oncology nurses: a pilot study. J Contin Educ Nurs.

[20] Balslev T, de Grave WS, Muijtjens AM, Scherpbier AJ (2005). Comparison of text and video cases in a postgraduate problem-based learning format. Med Educ.

[21] Kong J, Li X, Wang Y, Sun W, Zhang J (2009). Effect of digital problem-based learning cases on student learning outcomes in ophthalmology courses. Arch Ophthalmol.

[22] Bowdish B, Chauvin SW, Kreisman N, Britt M (2003). Travels towards problem based learning in medical education (VPBL). Instr Sci.

[23] Jin J, Bridges SM (2014). Educational technologies in problem-based learning in health sciences education: a systematic review. J Med Internet Res.

[24] Clark CE (2006). Problem-based learning: how do the outcomes compare with traditional teaching?. Br J Gen Pract.

[25] McParland M, Noble LM, Livingston G (2004). The effectiveness of problem-based learning compared to traditional teaching in undergraduate psychiatry. Med Educ.

[26] Rich SK, Keim RG, Shuler CF (2005). Problem-based learning versus a traditional educational methodology: a comparison of preclinical and clinical periodontics performance. J Dent Educ.

[27] Verstegen DML, de Jong N, van Berlo J, Camp A, Könings KD, van Merriënboer JJG, Donkers J, Bridges S, Chan LK, Hmelo-Silver CE (2016). How e-Learning Can Support PBL Groups: A Literature Review. Educational Technologies in Medical and Health Sciences Education.

[28] Higgins J, Green S (2011). Cochrane Handbook for Systematic Reviews of Interventions Version 5.1.

[29] Car J, Carlstedt-Duke J, Tudor Car L, Posadzki P, Whiting P, Zary N, Atun R, Majeed A, Campbell J, Digital Health Education Collaboration (2019). Digital Education in Health Professions: The Need for Overarching Evidence Synthesis. J Med Internet Res.

[30] (2013). United Nations Statistics Division.

[31] Li J, Li QL, Li J, Chen ML, Xie HF, Li YP, Chen X (2013). Comparison of three problem-based learning conditions (real patients, digital and paper) with lecture-based learning in a dermatology course: a prospective randomized study from China. Med Teach.

[32] Schmidt HG (1983). Problem-based learning: rationale and description. Med Educ.

[33] Laurillard D (2012). Teaching as a Design Science.

[34] Til Cv, Heijden FvD (2009). PBL Study Skills - an overview. Universiteit Maastricht.

[35] Cook DA, Hatala R, Brydges R, Zendejas B, Szostek JH, Wang AT, Erwin PJ, Hamstra SJ (2011). Technology-enhanced simulation for health professions education: a systematic review and meta-analysis. JAMA.

[36] Iniesta M, Alonso B, de Arriba L, Sanz M, Herrera D (2015). Enhancing learning with information and communication technology.

[37] Meyer KE (2011). ERIC.

[38] Heissam K, Sandokji A, El-Badry A (2010). The efficacy of web problem based learning versus traditional discipline learning in biomedical sciences. https://library.iated.org/view/HEISSAM2010THE.

[39] Dennis JK (2003). Problem-based learning in online vs. face-to-face environments. Educ Health (Abingdon).

[40] Taradi SK, Taradi M, Radic K, Pokrajac N (2005). Blending problem-based learning with Web technology positively impacts student learning outcomes in acid-base physiology. Adv Physiol Educ.

[41] Schutte B, de Goeij T, de Grave W, Koehorst AM, Scherpbier AJJA, van der Vleuten CPM, Rethans JJ, van der Steeg AFW (1997). The effects of visual genetics on the learning of students in a problem based curriculum. Advances in Medical Education.

[42] Sobocan M, Turk N, Dinevski D, Hojs R, Pecovnik Balon B (2017). Problem-based learning in internal medicine: virtual patients or paper-based problems?. Intern Med J.

[43] Moeller S, Spitzer K, Spreckelsen C (2010). How to configure blended problem based learning-results of a randomized trial. Med Teach.

[44] Alverson DC, Saiki SM Jr, Kalishman S, Lindberg M, Mennin S, Mines J, Serna L, Summers K, Jacobs J, Lozanoff S, Lozanoff B, Saland L, Mitchell S, Umland B, Greene G, Buchanan HS, Keep M, Wilks D, Wax DS, Coulter R, Goldsmith TE, Caudell TP (2008). Medical students learn over distance using virtual reality simulation. Simul Healthc.

[45] Wang J, Xu Y, Liu X, Xiong W, Xie J, Zhao J (2016). Assessing the effectiveness of problem-based learning in physical diagnostics education in China: a meta-analysis. Sci Rep.

[46] Zahid MA, Varghese R, Mohammed AM, Ayed AK (2016). Comparison of the problem based learning-driven with the traditional didactic-lecture-based curricula. Int J Med Educ.

[47] Qin Y, Wang Y, Floden RE (2016). The Effect of Problem-Based Learning on Improvement of the Medical Educational Environment: A Systematic Review and Meta-Analysis. Med Princ Pract.

[48] Sayyah M, Shirbandi K, Saki-Malehi A, Rahim F (2017). Use of a problem-based learning teaching model for undergraduate medical and nursing education: a systematic review and meta-analysis. Adv Med Educ Pract.

[49] Kong LN, Qin B, Zhou YQ, Mou SY, Gao HM (2014). The effectiveness of problem-based learning on development of nursing students' critical thinking: a systematic review and meta-analysis. Int J Nurs Stud.

[50] Sullivan GM (2011). Getting off the "gold standard": randomized controlled trials and education research. J Grad Med Educ.

