# Supplementary material for: Digital Problem-Based Learning in Health Professions: Systematic Review and Meta-Analysis by the Digital Health Education Collaboration
Source: J Med Internet Res. 2019 Feb 28;21(2):e12945. doi: 10.2196/12945 (PMC6416535; doi:10.2196/12945)
Supplement: Multimedia Appendix 1 [file jmir_v21i2e12945_app1.pdf]

Multimedia Appendix 1: Description of the digital education modalities

**Digital Leaning modality**	**Definitions/Description**
Offline computer-based learning (Offline digital education)	Offline digital education requires no internet or local area network connection and can be delivered through media including CD-ROM[a], external hard disc, and USB[b] stick [5].
Online computer-based learning (Online digital education)	Online digital education can be defined as those that require the use of a "Transmission Control Protocol" and an "Internet Protocol" as a standard for the learning activities. Alternatively, these may also be referred to as being 'online', 'web-based', or 'networked' [15].
Serious gaming and gamification (SGG)	Serious gaming and gamification is a competitive activity in which learners are set educational goals intended to promote knowledge acquisition. The games may either be designed to promote learning or the development of cognitive skills, or else take the form of simulations allowing learners to practice their skills in a virtual environment [45].
Massive open online course (MOOC)	A massive open online course is an online course that is designed for the participation of large numbers of geographically dispersed learners [46].
Virtual Reality (VR)	VR as the computer-generated representation of a real or artificial environment that can be interacted with by external involvement, allowing for a first-person active learning experience through immersion. We define 'immersive VR' as "complex technologies that replace real-world sensory information with synthetic stimuli such as 3D[c] visual imagery, spatialized sound, and force or tactile feedback" [6].
Virtual Patient (VP)	Virtual patients can be defined as "interactive computer simulations of real-life clinical scenarios for the purpose of medical training, education, or assessment" [47].
Psychomotor skills trainers (PST)	PST involves training procedure skills that includes "mental and motor activities required to execute a manual task" [48].
Mobile learning (mLearning)	mLearning was defined as "learning across multiple contexts, through social and content interactions, using personal electronic devices" [49].

[a]Compact Disc Read-Only Memory, [b]Universal Serial Bus, [c]Three-dimensional
