# Supplementary material for: Digital Problem-Based Learning in Health Professions: Systematic Review and Meta-Analysis by the Digital Health Education Collaboration
Source: J Med Internet Res. 2019 Feb 28;21(2):e12945. doi: 10.2196/12945 (PMC6416535; doi:10.2196/12945)
Supplement: Multimedia Appendix 3 [file jmir_v21i2e12945_app3.pdf]

Multimedia Appendix 3: Digital technologies used in the studies and associated learning types

**Study ID**	**Digital Technologies used in the study**	**Associated learning types**
Alverson *et al.* 2008 [44]	PBL[a] with digital presentation of problem and digitally delivered, distance-based PBL: Immersive Virtual Reality (VR) simulation-presented clinical with (out) remote communication using a multipoint videoconferencing and information sharing capability.	Acquisition, Collaboration, Discussion, Investigation, Practice, Production
Bowdish *et al.* 2003 [22]	PBL with digital presentation of problem: Interactive Web based virtual patient case scenarios consisting of videos, images, text, questions, radio button prompts and text boxes for posting group responses were used to simulate authentic scenarios and actions of everyday medical practice and patient care.	Acquisition, Practice
Dennis 2003 [39]	Digitally delivered, distance-based PBL: Web-based environment (including bulletin boards, chat rooms, and emails) was used to present the problem, interact with the facilitators, interact with colleagues and hold both meetings.	Acquisition, Collaboration, Discussion, Investigation, Production
Kong *et al.* 2009 [21]	PBL with digital presentation of problem: Digital cases were created out of videos of simulated patient scenarios.	Acquisition
Li *et al.* 2013 [31]	PBL with digital presentation of problem: Digital-form clinical cases in form of computer-based videos, presentations and photography of clinical cases	Acquisition
Moeller *et al.* 2010 [43]	Digitally delivered, distance-based learning: First part of the PBL (i.e. first-meeting) was delivered on a distance using a virtual workspace for presentation of case vignettes, collaboration and interaction using tools such as Wiki, Chat, and, Interactive Diagnostic Context (a diagnostic related link collection to expert sources). The rest of PBL was delivered face-to-face.	Acquisition, Collaboration, Discussion, Investigation
Schutte *et al.* 1997 [41]	PBL with digitally supported self-learning: A commercial 3D[b] software programme with 3D images of DNA structure and mRNA[c] synthesis was used to supplement the self-learning phase between the two PBL meetings.	Acquisition
Sobocan *et al.* 2017 [42]	PBL with digital presentation of problem: digitally enhanced, interactive, decision-making PBL in form of virtual patients (VP).	Acquisition, Practice
Taradi 2005 [40]	Digitally delivered, distance-based PBL: Web-based environment (including web pages, drawings, photos, flash animations, self-tests, bulletin boards, chat rooms, and emails) used to present the problem and interact with the facilitators and colleagues.	Acquisition, Collaboration, Discussion, Investigation, Production

[a]Problem-based learning, [b]Three-dimensional, [c]mRNA = messenger ribonucleic acid
