# Supplementary material for: Digital Problem-Based Learning in Health Professions: Systematic Review and Meta-Analysis by the Digital Health Education Collaboration
Source: J Med Internet Res. 2019 Feb 28;21(2):e12945. doi: 10.2196/12945 (PMC6416535; doi:10.2196/12945)
Supplement: Multimedia Appendix 4 [file jmir_v21i2e12945_app4.pdf]

Multimedia Appendix 4: Characteristics and effects of the included studies

**Study author, Design, Country**	**Comparison of Learning modality**	**Outcome assessments**	**Number and types of Participants**	**Field of the Study**	**Effect Sizes (based on SMD)[a]**	**Main conclusion**
DPBL[b] vs Traditional PBL[c]						
Alverson *et al.* 2008 [44], RCT[d], USA	VR[e] PBL vs Traditional PBL	Post-test mean score on questionnaires (Likert type)	36 Medical students (year unspecified)	Traumatic head injury	Not estimable	Knowledge: Not estimable
Bowdish *et al.* 2003 [22], RCT, USA	Online PBL vs Traditional PBL	Post-test mean score on Teaching and Learning Environment Questionnaire	150, Medical students (first year)	Human physiology	Knowledge: 0.04 [-0.33, 0.41]	Knowledge: DPBL = Traditional PBL
Dennis 2003 [39], RCT, USA	Online PBL vs Traditional PBL	Post-test mean score on MCQ[f] (30 items)	34, medical students (second year)	Pregnancy associated urinary incontinence	Knowledge: 0.28 [-0.40, 0.95]	Knowledge: DPBL = Traditional PBL
Kong 2009 *et al.* [21], RCT, China	Online PBL vs traditional PBL	Post-test mean score on questionnaires	90, Medical students (year unspecified)	Ophthalmology	Knowledge: 0.05 [-0.46, 0.55]	Knowledge: DPBL = Traditional PBL
Li 2013 *et al.* [31], RCT, China	Offline PBL vs Traditional PBL	Post-test mean score on both MCQ (20 items) and Likert scale (5 points)	120, Medical students (fourth year)	Dermatology	Knowledge: 0.05 [-0.46, 0.55] Skills: 0.37 [-0.14, 0.88]	Knowledge: DPBL = Traditional PBL Skills: DPBL = Traditional PBL

Moeller 2010 *et al.* [43], RCT, Germany	Online PBL vs Traditional PBL	Self-test mean score and Questionnaires	237, Medical students (year unspecified)	Multidisciplinary	Knowledge: 0.14 [-0.12, 0.40] Skills: 0.29 [0.03, 0.55]	Knowledge: DPBL = Traditional PBL Skills: DPBL > Traditional PBL
Sobocan 2017 *et al.* [42], RCT, Slovenia	VP[g]-based PBL vs Traditional PBL	Post-test mean score on exam test	34, Medical students (third year)	Internal medicine	Knowledge: -0.07 [-0.74, 0.60]	Knowledge: DPBL = Traditional PBL
Taradi 2005 *et al.* [40], RCT, Croatia	Online PBL vs Traditional PBL	Questionnaires	121, Medical students (second year)	Biochemistry (acid-base physiology)	Knowledge: 0.68 [0.28, 1.07]	Knowledge: DPBL > Traditional PBL
DPBL vs traditional learning						
Kong 2009 *et al.* [21], RCT, China	Online PBL vs Traditional Learning (lecture)	Post-test mean score on questionnaires	90, Medical students (year unspecified)	Ophthalmology	Knowledge: 0.9 [0.36, 1.43]	Knowledge: DPBL > Traditional Learning
Li 2013 *et al.* [31], RCT, China	Offline PBL vs Traditional Learning (lecture)	Post-test mean score on both MCQ (20 items) and Questions (Essays)	120, Medical students (fourth year)	Dermatology	Knowledge: 0.98 [0.44, 1.51] Skills: 1.13 [0.58, 1.67]	Knowledge: DPBL > Traditional Learning Skills: DPBL > Traditional Learning
Schutte *et al.* 1997 [41], RCT, The Netherlands	VR PBL vs Traditional Learning (textbook)	Post-test mean score on questions (28 closed and 5 open questions)	68, Medical student (first year)	Genetics (Global Structure of DNA)	Knowledge: 0.16 [-0.31, 0.64]	Knowledge: DPBL = Traditional Learning

[a]Standardized Mean Difference (SMD) values were based on Revman 5.3 calculator, Cochrane Collaboration, [b]Digital Problem Based Learning, [c]Problem Based Learning, [d]Randomized Controlled Trial, [e]Virtual Reality, [f]Multiple Choice Questions, [g]Virtual Patient
